# Comparison of ^68^Ga-PSMA-617 PET/CT with mpMRI for the detection of PCa in patients with a PSA level of 4–20 ng/ml before the initial biopsy

**DOI:** 10.1038/s41598-020-67385-9

**Published:** 2020-07-03

**Authors:** Yu Li, Donghui Han, Peng Wu, Jing Ren, Shuaijun Ma, Jingliang Zhang, Wei Song, Xiaoyu Lin, Dian Jiao, Shengjia Shi, Fa Yang, Jieheng Wu, Ping Meng, Weihong Wen, Fei Kang, Jing Wang, Weijun Qin

**Affiliations:** 1 Department of Urology, Xijing Hospital, Fourth Military Medical University, 127 West Changle Road, Xi’an, 710032 Shaanxi China; 2 Department of Nuclear Medicine, Xijing Hospital, Fourth Military Medical University, 127 West Changle Road, Xi’an, 710032 Shaanxi China; 3 Department of Radiology, Xijing Hospital, Fourth Military Medical University, Xi’an, China; 4 Department of Immunology, Fourth Military Medical University, Xi’an, China; 5 Institute of Medical Research, Northwestern Polytechnical University, Xi’an, China

**Keywords:** Urological cancer, Prostate, Cancer imaging

## Abstract

The study was aimed at assessing the diagnostic performance of ^68^Ga-PSMA-617 PET/CT in the detection of prostate cancer (PCa) in patients with a prostate-specific antigen (PSA) level of 4–20 ng/ml and to compare its efficacy with that of multiparametric MRI (mpMRI). We analyzed the data of 67 consecutive patients with PSA levels of 4–20 ng/ml who almost simultaneously underwent ^68^Ga-PSMA-617 PET/CT and mpMRI. ^68^Ga-PSMA-617 PET/CT and mpMRI diagnostic performances were compared via receiver operating characteristic (ROC) curve analysis. Of the 67 suspected PCa cases, 33 had pathologically confirmed PCa. ^68^Ga-PSMA-617 PET/CT showed a patient-based sensitivity, specificity, and positive and negative predictive values (PPVs and NPVs) of 87.88%, 88.24%, 87.88%, and 88.24%, respectively. The corresponding values for mpMRI were 84.85%, 52.94%, 63.64%, and 78.26%. The area under the curve values for ^68^Ga-PSMA-617 PET/CT and mpMRI were 0.881 and 0.689, respectively. ^68^Ga-PSMA-617 PET/CT showed a better diagnostic performance than mpMRI in the detection of PCa in patients with PSA levels of 4–20 ng/ml.

## Introduction

Prostate cancer (PCa) is the second-most prevalent malignancy in men and the fifth leading cause of cancer-related death worldwide^1^. Based on the Surveillance Epidemiology and End Results data, the 5-year survival for local or regional PCa is effectively 100% but decreases to 34% in cases with distant metastases^2^. Therefore, the early detection and identification of PCa are critical in decision-making for optimal treatment ^3^.

Prostate-specific antigen (PSA) screening has been most commonly used for early PCa detection. When the PSA level is equal to or higher than 20 ng/ml, the serum PSA test is up to 87.2% accurate in predicting PCa^4^. However, owing to the organ specificity rather than the tumor specificity of PSA, PSA screening still has some limitations in detection of PCa and may cause overdetection^5^. The most prominent diagnostic difficulty associated with PSA screening is the detection of PCa in patients with PSA levels of 4–20 ng/ml, which is mainly reflected by relatively high false-positive and false-negative rates. It was reported that in patients with PSA levels of < 20 ng/ml, the detection rate of PSA screening were less than 36% and the specificity was only approximately 10.27%^6^. In other words, the false-positive rate was up to 89.73%. The high false-positive rate of PSA screening may lead to unnecessary repeated prostate biopsies to verify or exclude the risk of malignancy, resulting in higher costs and adverse effects, such as infection, bleeding, and tissue damage. Therefore, it is essential to determine other diagnostic methods to improve the diagnostic performance for PCa in patients with PSA levels of 4–20 ng/ml.

Multiparametric magnetic resonance imaging (mpMRI) is a modern non-invasive diagnostic tool for PCa detection, with high sensitivity and specificity, affording an improved diagnostic performance^7^. However, the results of some studies evaluating the diagnostic performance of mpMRI are controversial. A systematic review and diagnostic meta-analysis reported that the pooled average sensitivity and specificity of mpMRI for PCa detection were 89% and 73%^8^. Although Thompson et al.^9^ and Kim et al.^10^ showed that mpMRI sensitivity for the detection of PCa was up to 96% while the specificity was only 36–58%, indicating a relatively high false-positive rate.

Positron emission tomography/computerized tomography (PET/CT) with ligands for the prostate-specific membrane antigen (PSMA) may overcome this limitation. PSMA expression is significantly higher in PCa cells than in normal prostatic tissue^11^. PSMA–Glu–NH–CO–NH–Lys(Ahx)-HBED-CC (PSMA-11) and PSMA–Glu–NH–CO–NH–Lys-2-naphthyl-l-Ala-cyclohexane-DOTA (PSMA-617) are both specific PSMA ligands, which have shown significantly improved binding affinity to PSMA and highly efficient internalization into PCa cells^12,13^. Labeled with gallium-^68^(^68^Ga), both radiotracers have shown good diagnostic performance for PCa^14,15^. Therefore, ^68^Ga-PSMA PET/CT is expected to be the potential solution to the diagnostic difficulty in the detection of PCa in patients with PSA levels of 4–20 ng/ml.

Therefore, our aim was to assess the diagnostic performance of ^68^Ga-PSMA-617 PET/CT in the detection of PCa in patients with PSA levels of 4–20 ng/ml and to compare its efficacy with that of mpMRI. Furthermore, we analyzed ^68^Ga-PSMA-617 PET/CT parameters such as maximal standardized uptake value (SUVmax) and SUVratio to determine the correlation between the parameter and clinical indicators such as PSA level and Gleason score (GS).

## Methods

### Patient characteristics

We analyzed the data of 115 consecutive patients who almost simultaneously underwent both ^68^Ga-PSMA-617 PET/CT and mpMRI from May 2017 to July 2018. The study was approved by the ethics committee (approval no. KY20162088-1) and conducted in the Urology Department, Radiology and Nuclear Medicine Department of Xijing Hospital. Patients with suspected PCa according to the National Comprehensive Cancer Network (NCCN) guidelines^16^ were included if they met the following requirements: age of 40–85 years; and (2) very suspicious digital rectal examination (DRE) results or total PSA (tPSA) of 4–20 ng/ml. The following patients were excluded: (1) patients who had received any PCa treatment such as androgen deprivation therapy, radiation therapy, chemotherapy, immunotherapy, or any type of prostate surgery before ^68^Ga-PSMA-617 PET/CT or mpMRI; and (2) patients for whom there was histologically proven diagnosis of PCa. Sixty-seven patients with sufficient clinical data were eligible for the analysis. All patients underwent transrectal ultrasound biopsy (TRUSB), and patients for whom the initial biopsy results were negative accepted further histological examination or at least a 1-year follow-up. All patients provided written informed consent for ^68^Ga-PSMA-617 PET/CT and biopsy, as well as anonymous publication of clinical data in this study. Table 1 summarizes the characteristics of the patients examined.

**Table 1:** Clinical and pathological characteristics of 67 patients investigated in this study.

Characteristics	Value or number of patients
**Age**	
Mean ± SD	68.91 ± 8.34
Median (range)	68 (42–85)
**tPSA**	
Mean ± SD	11.00 ± 4.99
Median (range)	10.48 (3.15–19.76)
**SUVmax**	
Mean ± SD	7.67 ± 7.19
Median (range)	4.30 (2.10–41.30)
**SUVratio**	
Mean ± SD	2.80 ± 2.08
Median (range)	1.84 (1.05–8.78)
**DRE (%)**	
Abnormal	37 (55.22)
Normal	30 (44.78)
**PI-RADS (%)**	
1–2	23 (34.33)
3	13 (19.40)
4	23 (34.33)
5	8 (11.94)
**Gleason score (%)**	
0	34 (50.75)
6	3 (4.48)
7	12 (17.91)
8	10 (14.93)
9	6 (8.96)
10	2 (2.99)
**ISUP grade (%)**	
0	34 (50.75)
1	3 (4.48)
2	4 (5.97)
3	8 (11.94)
4	10 (14.93)
5	8 (11.94)

### ^68^Ga-PSMA-617 PET/CT

^68^Ga-PSMA-617 PET/CT was performed on Biograph 40 PET/CT scanner (Siemens, Germany), ^68^Ge/^68^Ga-generator was from ITG company (Germany), and PSMA-617 ligand was from ABX company (Germany). ^68^Ga-PSMA-617 was prepared according to a previously published method^17^ with a radiochemical purity of > 95%. The ^68^Ga-PSMA-617 prepared was administered via elbow vein injection at a dose of 3–5 mCi. Whole-body PET scans were acquired approximately 60 min after tracer injection. No adverse or clinically detectable pharmacological effects were observed in any of the enrolled patients.

### mpMRI

mpMRI was performed on a 3.0-T MR scanner (Achieva 3.0 T TX, Philips Medical Systems, The Netherlands) by using a 16-channel phased-array coil. Transverse/coronal/sagittal (18 slices, thickness: 3 mm, gap: 0.5 mm, TR: 3,744 ms, TE: 120 ms, number of signals acquired: 2, resolution: 1.49 mm × 1.51 mm) T2-weighted turbo spin-echo images were acquired. Diffusion-weighted imaging (DWI) with spin-echo echo-planar images (18 slices, thickness: 3 mm, intersection gap: 1 mm, TR: 925 ms, TE: 41 ms, number of signals acquired: 1, resolution: 3 mm × 3 mm, b-factor: 0/800/1,500 s/mm^2^) were acquired. T1 high-resolution isotropic volume with fat suppression after gadolinium injection was employed for dynamic enhanced (DCE) images (133 slices, thickness: 3 mm, intersection gap: none, TR: 3.1 ms, TE: 1.46 ms, number of signals acquired: 1, resolution: 1.49 mm × 1.51 mm, dynamic scan time: 00:06.9). Mappings of the ADC were generated from b 0, b 800, and b 1,500 images of DWI using Philips WorkStation software (Extended Workspace, EWS). The technique and operation parameters of mpMRI were based on previous research by Professor Jing Ren^18^ and the current clinical practice of the department of radiology, Xijing Hospital.

### Imaging analysis

The ^68^Ga-PSMA-617 PET/CT images were reviewed by consensus between three experienced nuclear medicine physicians by using Siemens MMWP workstation. PCa lesions were distinguished from the surrounding normal prostate tissues by visual analysis. To calculate the ^68^Ga-PSMA-617 uptake of the primary PCa lesion, circular regions of interest (ROIs) were drawn around areas with focally increased uptake in transaxial slices by using e.soft software (Siemens) at a 30% threshold, and SUVmax was calculated automatically. Scans were considered positive when the focal uptake of ^68^Ga-PSMA-617 was superior to the background activity. Semiquantitative measures comprised SUVmax and SUVratio (explained below under “Statistical analysis”).

The positive criterion for mpMRI is an abnormal focal signal increase or decrease. The readers were instructed to decide on the basis of their overall impression by using the 5-point Prostate Imaging Reporting and Data System (PI-RADS) score^19^: 1, PCa highly unlikely; 2, PCa unlikely; 3, equivocal PCa; 4, PCa likely; and 5, PCa highly likely. At the patient level, data were dichotomized (score of 1–2, PCa negative; score of 3–5, PCa positive) using the highest score per patient.

### Histological examination

After PET/CT and mpMRI scans, all 67 patients underwent TRUSB. According to the NCCN Clinical Practice Guideline in PCa^16,20^, repeated saturation biopsy (≥ 18 cores) was performed in negative cases during the follow-up to avoid a potential false-negative diagnosis in the initial biopsy. A diagnosis of malignancy was defined as a positive biopsy result. Benign diagnoses were comprehensively defined as repeated negative pathological findings on biopsies and continuous follow-up by monthly PSA monitoring, imaging-based monitoring (mpMRI or ^68^Ga-PSMA-617), and clinical symptom examination for at least 1 year. Subsequently, PCa patients who met the indications for surgery received robot-assisted laparoscopic radical prostatectomy (RALRP) and prostatectomy specimens were obtained. The biopsy tissues and specimens were formalin-fixed and routinely processed by hematoxylin–eosin staining and immunohistochemistry (IHC) analysis as previously reported^21^. Histopathological results of the biopsy tissues served as a reference for PCa diagnosis, which was stratified according to the 8th edition of the AJCC staging system for PCa^22^.

### Statistical analysis

Continuous variables are presented as means ± standard deviations or medians (interquartile ranges). Categorical variables are presented as frequencies (percentages). The number of PCa cases determined using ^68^Ga-PSMA-617 and mpMRI were counted, and the diagnostic performance of ^68^Ga-PSMA-617 PET/CT and mpMRI was evaluated. The overall diagnostic accuracy of the two methods by using patient-level data was assessed by receiver operating characteristic curve (ROC) analysis. Areas under the ROC curves (AUCs) with 95% confidence intervals (CIs) were compared to each other as well as to a fixed value of 0.5. SUVratio was determined by dividing the highest SUVmax in PCa lesions by the SUVmax of the background (maximal uptake in the normal tissues surrounding the prostate gland), which reflected the imaging contrast of ^68^Ga-PSMA-617 PET/CT. We used one-way ANOVA and Student's *t* test to evaluate the relationships between various SUVmax or SUVratio measurements and clinical parameters such as age, PSA value, and GS. P values < 0.05 were considered statistically significant. Correlation analysis was assessed using the Spearman rank correlation coefficient. The provided boxplots show the first and third quartiles and median. The ends of the whiskers represent the 10th and 90th percentiles. The data were analyzed using IBM SPSS software, version 24.0 (IBM, Inc., Chicago, IL, USA), GraphPad Prism software, version 7.0 (GraphPad Software, Inc., La Jolla, CA, USA), and R software, version 3.4.2 (R Foundation for Statistical Computing, Vienna, Austria).

## Results

### Patient characteristics

**Figure 1:**
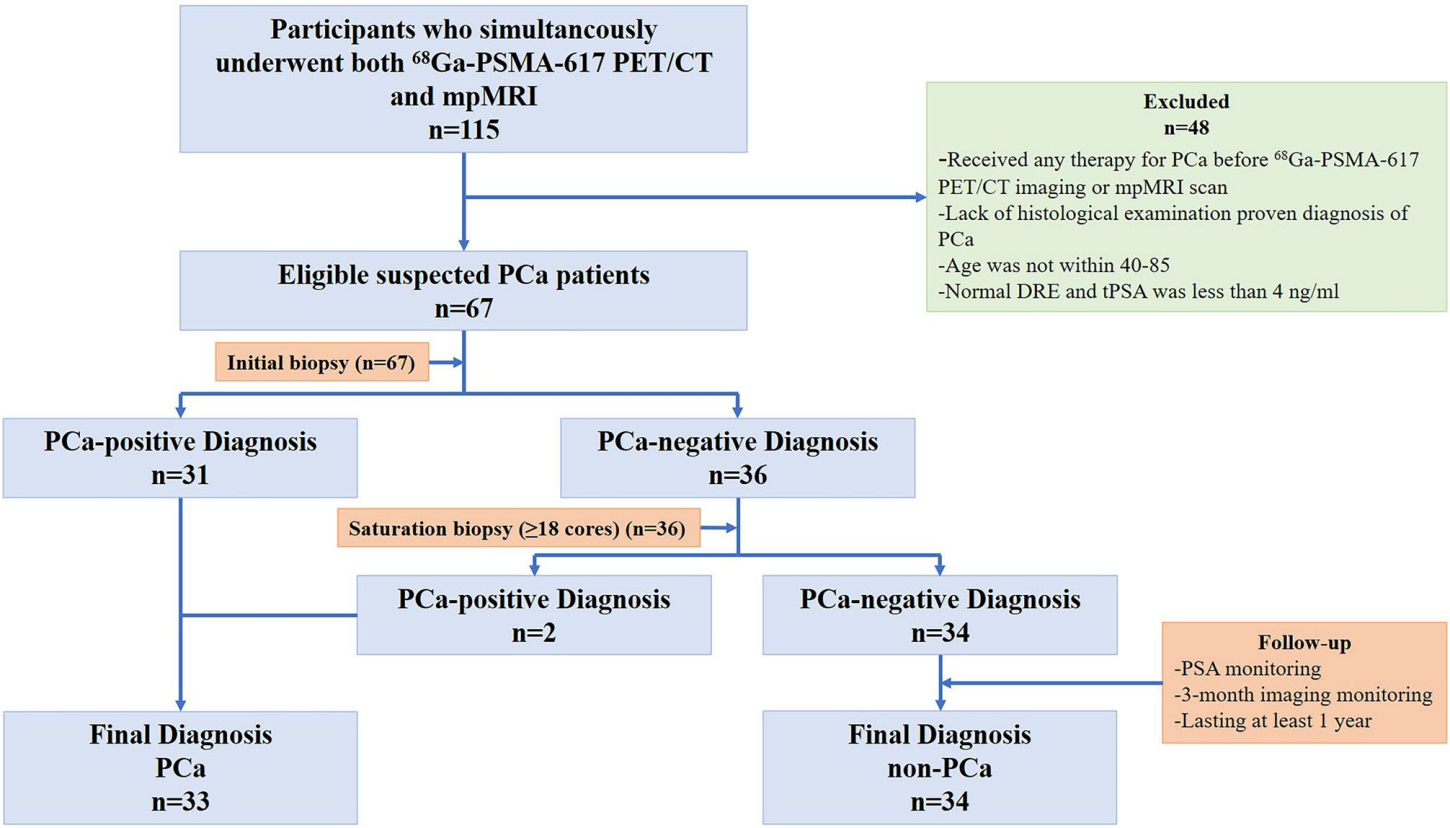
Procedure used for pathological diagnosis in the study.

In this study, 115 men underwent ^68^Ga-PSMA-617 PET/CT and mpMRI, among whom 67 patients were included in the final analysis, according to the inclusion and exclusion criteria shown in Fig. 1. The mean time between the two examinations (^68^Ga-PSMA-617 PET/CT and mpMRI) and the histopathological examination was 17.5 days (range 1–56 days) and 15.64 days (1–35 days), respectively.

The overall characteristics of the 67 patients are presented in Table 1. Based on the results of the initial biopsy, 31 patients were PCa-positive and 36 were PCa-negative. The 36 PCa-negative patients underwent a secondary saturation biopsy (≥ 18 cores) 6–8 weeks later, and the pathology results suggested that two patients had PCa. The remaining 34 patients were followed-up for at least 1 year. The follow-up included serum PSA monitoring per month and 3-month imaging monitoring (mpMRI or ^68^Ga-PSMA-617). The follow-up examination showed no PSA or imaging progression in any of the 34 patients who were defined as showing benign disease according to the NCCN Clinical Practice Guideline in PCa^16,20^. Of the 33 patients who were already diagnosed as having PCa, 21 had surgical indications, and whole-mount specimens were obtained after RALRP for further immunohistochemical analysis of PSMA espression.

### Diagnostic performance of ^68^Ga-PSMA-617 PET/CT and mpMRI

**Figure 2:**
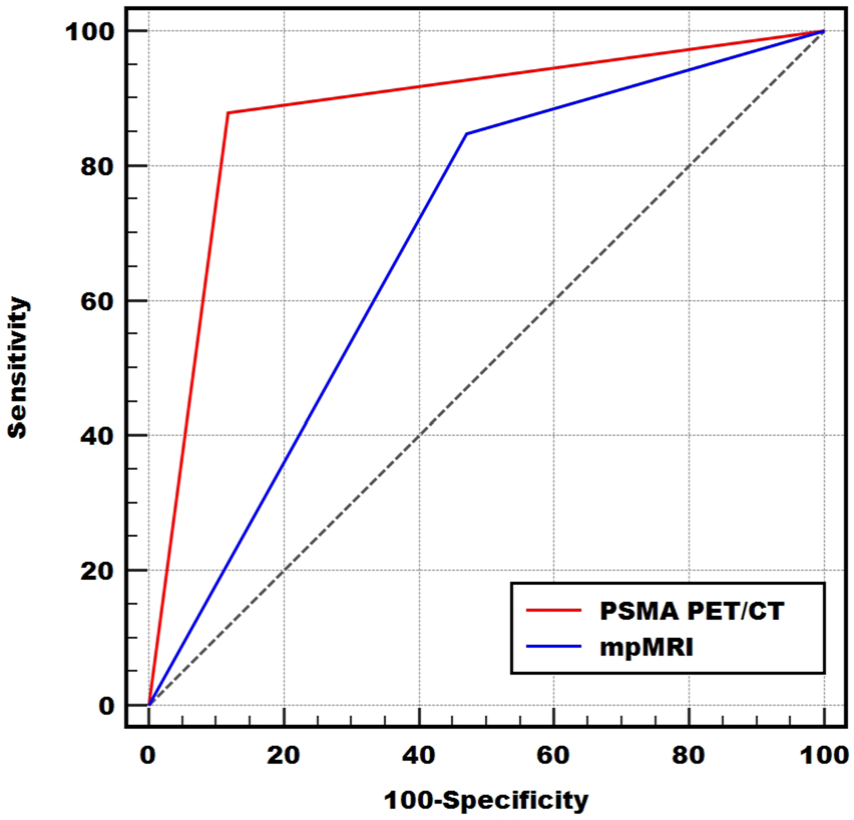
Receiver operating characteristic (ROC) curves of ^68^Ga-PSMA-617 PET/CT and mpMRI for detection of PCa.

**Figure 3:**
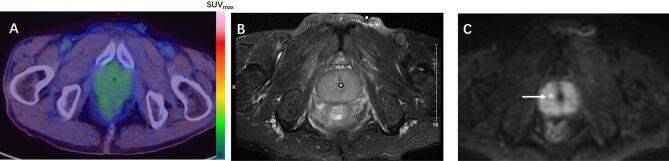
A 73-year-old patient with a PSA level of 4.29 ng/ml. The biopsy results of the two punctures showed chronic granulomatous inflammation of the prostate tissue. After 3 months of follow-up, the PSA fluctuations ranged from 3.52 to 5.39 ng/ml, suggesting benign lesions. ^68^Ga-PSMA-617 PET/CT (**A**) showed no significant change in the uptake of ^68^Ga-PSMA-617 in the prostate, with an SUVmax of 3.0, consistent with benign prostatic lesions. In mpMRI, there was no obvious abnormal signal on T2W (**B**), but DWI (**C**) suggested indicated a spot abnormal signal in the center of the right side of the prostate (indicated by the arrow), suggesting PCa, but it was inconsistent with the pathological results.

As shown in Table 2, among the 33 patients with confirmed PCa, 28 were positive on mpMRI and five were negative. ^68^Ga-PSMA-617 PET/CT showed that 29 patients were positive and four were negative. Of the 34 patients with benign diagnoses, 16 were positive on mpMRI and 18 were negative. ^68^Ga-PSMA-617 PET/CT showed that four patients were positive and 30 were negative. The patient-based sensitivity, specificity, PPV, and NPV of ^68^Ga-PSMA-617 PET/CT were 87.88% (29/33, 95% CI 80.86–96.04%), 88.24% (30/34, 95% CI 71.61–96.16%), 87.88% (29/33, 95% CI 70.86–96.04), and 88.24% (30/34, 95% CI 71.61–96.16%) respectively. The corresponding values for mpMRI were 84.85% (28/33, 95% CI 67.33–94.28), 52.94% (18/34, 95% CI 35.40–69.84), 63.64% (28/44, 95% Cl 47.74–77.17%), and 78.26% (18/23, 95% CI 55.79–91.71%) (Table 3). We performed an ROC analysis for the two examinations and compared the results at a patient-level (Fig. 2). The AUC values of ^68^Ga-PSMA-617 PET/CT and mpMRI were 0.881 (95% CI 0.778–0.947) and 0.689 (95% CI 0.564–0.796) respectively, which indicated that ^68^Ga-PSMA-617 PET/CT may have a better diagnostic performance (*P* = *0.0019*). SI Figure S1 shows a representative case in which the primary lesion could be detected by both ^68^Ga-PSMA-617 PET/CT and mpMRI. Figure 3 shows a representative case in which ^68^Ga-PSMA-617 PET/CT could correct false-positive errors of mpMRI with superior specificity.

**Table 2:** Diagnostic test evaluation results of mpMRI and ^68^Ga-PSMA-617 PET/CT.

	PCa	Non-PCa	Total
**mpMRI**			
MRI ( +)	28	16	44
MRI (−)	5	18	23
Total	33	34	67
^**68**^**Ga-PSMA-617**			
PET/CT ( +)	29	4	33
PET/CT (−)	4	30	34
Total	33	34	67

**Table 3:** Diagnostic performance of ^68^Ga-PSMA-617 PET/CT and mpMRI in the detection of prostate cancer.

	Sensitivity (95% CI)	Specificity (95% CI)	PPV (95% CI)	NPV (95% CI)	AUC (95% CI)	*P*
^68^Ga-PSMA-617 PET/CT	87.88% (80.86–96.04)	88.24% (71.61–96.16)	87.88% (70.86–96.04)	88.24% (71.61–96.16)	0.881 (0.778–0.947)	0.0019
mpMRI	84.85% (67.33–94.28)	52.94% (35.40–69.84)	63.64% (47.74–77.17)	78.26% (55.79–91.71)	0.689 (0.564–0.796)	

*PPV* positive predictive value, *NPV* negative predictive value, *AUC* area under the curve, *CI* confidence interval.

### Comparison of SUVmax, SUVratio, and PI-RADS score with clinical parameters

The ROI uptake values on ^68^Ga-PSMA PET/CT were as follows: median SUVmax, 4.30 (range 2.10–41.30); median background SUV, 2.27 (range 1.51–5.29); and SUVratio, 1.84 (range 1.05–8.78). As shown in Table 4 and SI Figure S2, in the 67 suspected patients, the SUVmax, SUVratio, and PI-RADS score of benign prostate tissue were lower than those of PCa tissue (*P1* < *0.0001*; *P2* < *0.0001; P3* = *0.0002,* respectively).

**Table 4:** Comparison of the uptake values of ^68^Ga-PSMA-617 PET/CT (SUVmax and SUVratio) and clinical parameters.

Clinical parameter	SUVmax	F1/t1	*P1*	SUVratio	F2/t2	*P2*	PI-RADS	K-W/K-S	*P3*
**Age**									
40–59	9.23 ± 13.08	0.55	0.6530	2.78 ± 2.24	0.29	0.8310	3.13 ± 1.13	0.10	0.9915
60–69	6.340 ± 5.62			2.54 ± 1.90			3.14 ± 1.18		
70–79	8.18 ± 6.22			3.09 ± 2.38			3.09 ± 1.24		
80–85	9.078 ± 7.78			2.90 ± 1.93			3.25 ± 1.58		
**PSA (ng/ml)**									
< 10	3.81 ± 2.16	4.31	< 0.0001	1.53 ± 0.45	5.11	< 0.0001	2.76 ± 1.15	0.39	0.0134
10–20	10.61 ± 8.26			3.77 ± 2.32			3.42 ± 1.20		
**Intraprostatic tissue**									
Normal	3.49 ± 1.20	5.97	< 0.0001	1.42 ± 0.31	7.42	< 0.0001	2.53 ± 1.16	0.52	0.0002
Malignant	11.98 ± 8.21			4.22 ± 2.68			3.76 ± 0.94		
**Gleason score**									
6	5.02 ± 1.17	8.08	0.0002	2.39 ± 0.40	3.21	0.0270	2.68 ± 1.16	9.43	0.0510
7	9.43 ± 5.51			3.55 ± 1.52			3.42 ± 0.79		
8	10.40 ± 6.95			4.00 ± 2.42			4.10 ± 0.88		
9	16.59 ± 3.08			6.09 ± 2.12			4.17 ± 0.75		
10	31.74 ± 13.52			6.54 ± 1.80			4.50 ± 0.71		
**Clinical significance**									
Non-csPCa	3.61 ± 1.25	6.57	< 0.0001	4.40 ± 2.20	7.88	< 0.0001	3.87 ± 0.86	0.55	< 0.0001
csPCa	12.67 ± 8.30			1.50 ± 0.41			2.54 ± 1.15		

*K–W* Kruskal–Wallis test, *K–S* Kolmogorov–Smirnov test.

The SUVmax, SUVratio, and PI-RADS score of different PSA groups showed significant differences (*P1* < *0.0001*, *P2* < *0.0001*, and *P3* = *0.0134*, respectively) (Table 4). Differences in SUVmax and SUVratio also remained statistically significant for the thee GS 6–10 groups (F1 = 8.075, *P1* = *0.0002*; F2 = 3.214, *P2* = *0.0273*, respectively) (Table 4; SI Figure S3). However, the PI-RADS score showed no significant difference in the GS 6–10 groups (*P3* = *0.0510*). On the basis of the previous results, we performed Spearman correlation analysis on the data, and the results showed that ^68^Ga-PSMA-617 uptake (SUVmax or SUVratio) was significantly positively correlated with the GS (r1 = 0.5510, *P1* = *0.0009*; r2 = 0.5058, *P2* = *0.0027*, respectively), while Pearson correlation analysis showed a similar positive correlation with PSA levels (r1 = 0.5064, *P1* < *0.0001*; r2 = 0.4924, *P2* < *0.0001*) (SI Figure S3).

With reference to the findings of the pathology examination, SI Figure S4 showed that both the ^68^Ga-PSMA-617 uptake values (SUVmax or SUVratio) and PI-RADS score were significantly higher in clinically significant PCa (GS ≥ 7) than in non-clinically significant PCa (GS = 6 or benign lesions) (*P* < *0.0001*) (Table 4; SI Figure S4).

### Semiquantitative analyses and optimal cut-off points of ROC curves: detection of PCa and clinically significant PCa

**Figure 4:**
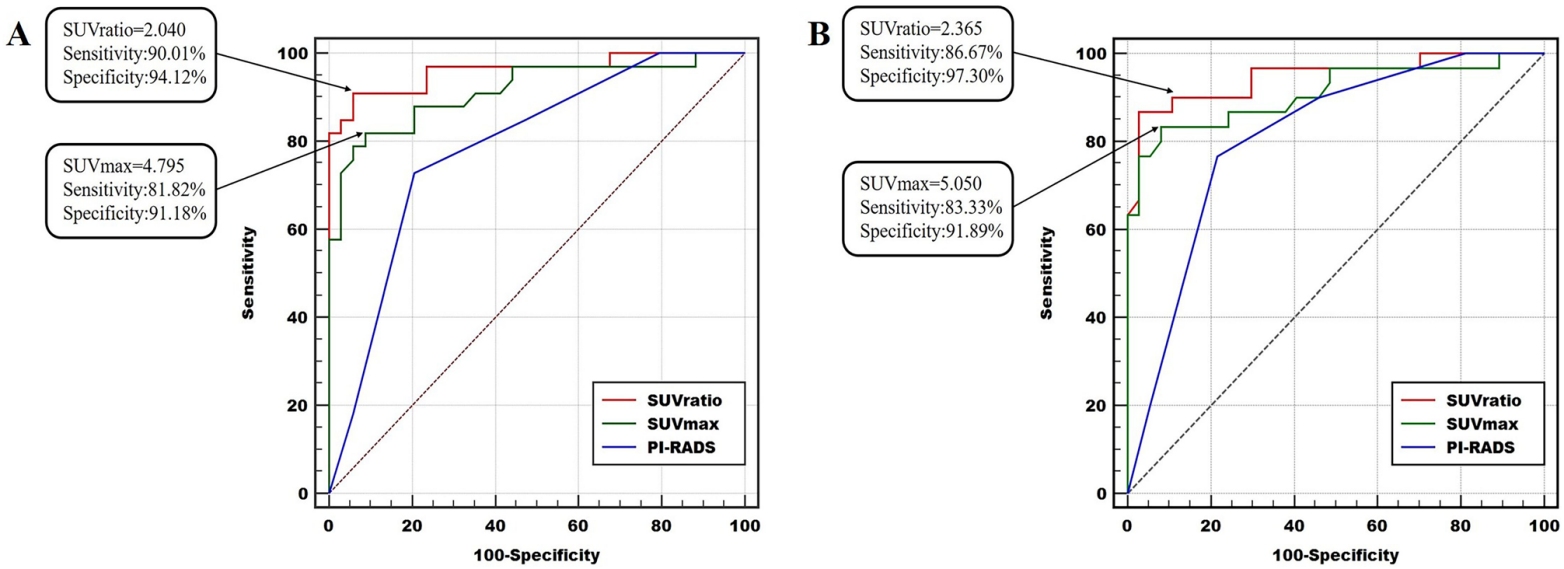
Receiver operating characteristic (ROC) curves of SUVmax, SUVratio, and PI-RADS for detecting PCa and clinically significant PCa with PSA levels of 4–20 ng/ml.

On ROC analysis (Fig. 4A), we determined the optimal cut-off points for SUVmax, SUVratio, and PI-RADS score according to the results of pathology examinations. We investigated the data by categorizing the lesions as benign (benign prostatic tissue) or malignant (any GS). The ROC analysis demonstrated that SUVmax > 4.795 and SUVratio > 2.040 could differentiate tumor lesions from benign conditions with sensitivities of 81.82% and 90.01% respectively, and specificity of 91.18% and 94.12% respectively, which indicated the largest AUCs (AUC1 = 0.9140, 95% CI 0.8429–0.9851; AUC2 = 0.9608, 95% CI 0.9145–1.000). The ROC analysis using statistical software demonstrated that when the PI-RADS score was 3.5, the AUC was the largest (AUC = 0.7857, 95% CI 0.6780–0.8933). When the PI-RADS score was selected as 3 or 4, the corresponding AUC was 0.6890 (95% CI 0.5640–0.7960) or 0.7610 (95% CI 0.6410–0.8570), and there was no significant difference between the two results (*P* = *0.1352*). According to Prostate Imaging Reporting and Data System Version 2^19^, the cut-off PI-RADS score was selected as 3, with AUC = 0.7210 (95% CI 0.6190–0.8090), which was significantly different from the ^68^Ga-PSMA-617 uptake values (SUVmax or SUVratio) (*P1* = *0.0427, P2* = *0.0013*).

We further investigated the data by dividing the lesions into non-clinically significant PCa (PCa with a Gleason score of 6 and benign prostatic tissue) versus clinically significant PCa (PCa with GS ≥ 7). The ROC analysis demonstrated that the optimal cut-off points were 5.050 (SUVmax) and 2.365 (SUVratio), which could differentiate tumor lesions from benign conditions with sensitivities of 83.33% and 86.67% respectively, and specificities of 91.89% and 97.30%, respectively, thereby indicating the largest AUC (AUC1 = 0.9086, 95% CI 0.8308–0.9864; AUC2 = 0.9477, 95% CI 0.8922–1) (Fig. 4B). The ROC analysis by statistical software demonstrated that when the PI-RADS score was 3.5, the AUC was the largest (AUC = 0.8095, 95% CI 0.7086–0.9103). When the PI-RADS score was 3 or 4, the corresponding AUC was 0.689 or 0.761, respectively, and there was no statistical difference between the two results (*P* = *0.2492*), while the SUVratio showed a significant difference *(P* = *0.0093)* and the SUVmax showed no statistical difference *(P* = *0.1164)*.

### Comparison of the clinical utility between ^68^Ga-PSMA-617 PET/CT and mpMRI

**Figure 5:**
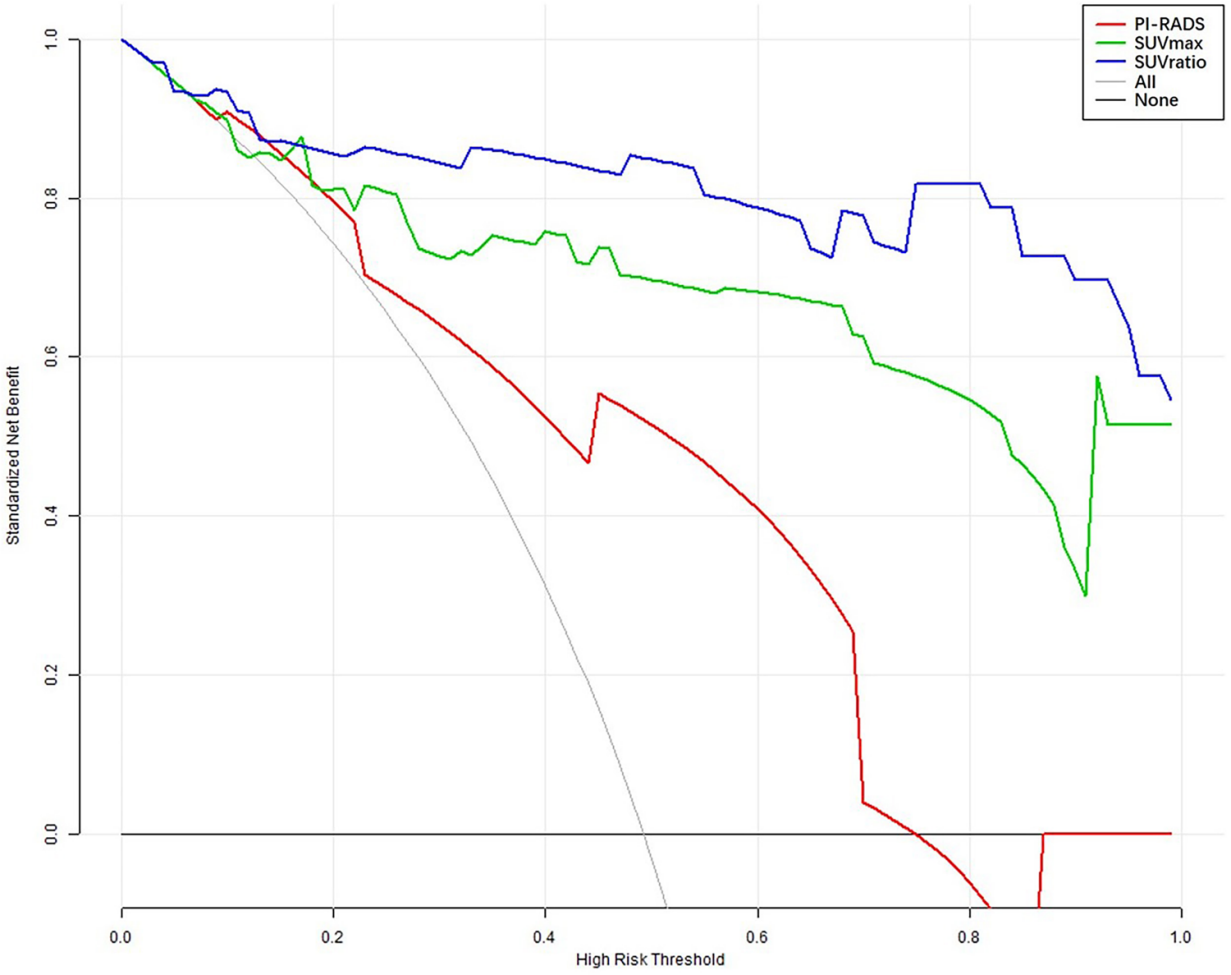
Decision curve analysis of the clinical utility of different diagnostic parameters of ^68^Ga-PSMA-617 PET/CT and mpMRI and risk calculators for the detection of PCa and clinically significant PCa with PSA levels of 4–20 ng/ml.

As shown in Fig. 5, to determine which diagnostic parameters of ^68^Ga-PSMA-617 PET/CT and mpMRI had better clinical utility in assisting biopsy decisions, we performed decision curve analysis (DCA) of SUVmax, SUVratio, and PI-RADS score. The grey line (leftmost) represents the “biopsy all patients” strategy, and the horizontal black line indicates the “biopsy none” strategy. Curves representing each diagnostic parameter are indicated. As expected, all of the methods were superior to the “biopsy all patients” strategy, and the net benefit of PI-RADS was the lowest of the three methods, while the net benefit of SUVratio was higher than that of SUVmax. The net benefit of SUVmax and SUVratio was greater for patients within threshold probabilities of 20–95% than PI-RADS, with the outcome as PCa (Fig. 5), suggesting that the clinical utility of ^68^Ga-PSMA-617 PET/CT was superior to that of mpMRI on most occasions. Hence, DCA showed that the SUVratio of ^68^Ga-PSMA-617 PET/CT might be the preferred diagnostic parameter in the detection of PCa with PSA levels of 4–20 ng/ml.

### Immunohistochemical analysis of the PSMA expression in PCa tissues

As shown in Table 4 and SI Figure S3, we have already confirmed the positive correlation between SUVmax and GS. SI Figure S5 intuitively showed this correlation. Furthermore, to evaluate the expression pattern of PSMA in PCa, we performed immunohistochemical staining in PCa tissues from 25 patients who agreed to provide tissue specimens. Among the 25 samples, one (4%) did not show detectable PSMA expression, while three (12%) samples showed PSMA expression in < 50% of PCa tissues, and in 21 (84%) samples, PSMA expression was > 50%.

## Discussion

To the best of our knowledge, this is the first study to evaluate the diagnostic performance of ^68^Ga-PSMA-617 PET/CT and mpMRI in the detection of PCa with PSA levels of 4–20 ng/ml. Our data support the hypothesis that ^68^Ga-PSMA-617 PET/CT can more accurately differentiate primary PCa with PSA levels of 4–20 ng/ml than mpMRI, and that ^68^Ga-PSMA-617 uptake is positively correlated with the clinical risk parameters GS and PSA level. Moreover, as an important parameter of ^68^Ga-PSMA-617 PET/CT, the SUVratio seems to be a superior predictive factor owing to its better discriminative ability to predict PCa, thereby improving diagnostic performance.

Considering the clinical difficulties in the diagnosis of PCa with PSA levels of 4–20 ng/ml, serum PSA screening might not seem to be an ideal method for the detection of PCa. Lojanapiwat et al. reported that when the cut-off PSA level was higher than 20 ng/ml, the specificity was significantly improved^23^. In comparison with traditional imaging studies, mpMRI is currently the most commonly used clinical imaging examination method, through T1, T2-weighted images, DWI, DCE, and other functional sequences. Multi-analysis imaging can better show the integrity of the prostate capsule and tumor invasion into tissues and organs around the prostate. MpMRI can also show invasion of pelvic lymph nodes and lesions of bone metastases, which may play important roles in clinical staging. Current guidelines propose the use of mpMRI for local staging in primary PCa^24^. However, clinical MRI examinations cannot simultaneously perform a whole-body scan and usually perform well for local conditions such as those limited to the chest, abdomen, pelvis, etc. Whole-body assessments using these examination methods have limitations, making the examination less comprehensive and the staging not accurate enough. In addition, it is difficult for elderly patients with PCa to remain motionless for a long time during the examination process, thus resulting in motion artifacts. Furthermore, metal implants such as pacemakers and artificial joints are contraindicated in this examination. In contrast, ^68^Ga-PSMA-617 PET/CT is a non-invasive and full-body imaging modality, with fewer contraindications and easier acceptance by patients^25^.

Our study systematically evaluated the potential of ^68^Ga-PSMA-617 PET/CT for the detection of primary PCa with PSA levels of 4–20 ng/ml, and the findings indicated the excellent diagnostic performance of ^68^Ga-PSMA-617 PET/CT in comparison with mpMRI. In terms of sensitivity, ^68^Ga-PSMA-617 PET/CT and MRI both showed good diagnostic performance (87.88% vs. 84.85%), while in terms of specificity, ^68^Ga-PSMA-617 PET/CT was better than mpMRI (88.24% vs. 52.94%). In their study based on histopathological segment analysis, Eiber et al.^26^ demonstrated that in comparison with mpMRI, ^68^Ga-PSMA PET/CT had higher diagnostic accuracy (AUC), sensitivity, and specificity for the detection of primary PCa (0.83 vs. 0.73, 92% vs. 66%, and 94% vs. 82%, respectively). Giesel et al.^27^, Scheltema et al.^28^, and Chen et al.^29^ also reported that ^68^Ga-PSMA PET/CT offers advantages over mpMRI for intra-prostatic tumour localization. These previous results were consistent with our present study in terms of the diagnostic performance of ^68^Ga-PSMA PET/CT; however, for mpMRI, our study showed a lower specificity. A possible reason for this discrepancy might be that the definition of PCa in our present study included GS ≥ 6 and the diagnostic selected threshold of PI-RADS was 3. When we only considered clinically significant PCa (GS ≥ 7) and chose PI-RADS 4 as the diagnostic selected threshold, the diagnostic accuracy (AUC) of mpMRI went up to 0.761, which was consistent with the previous studies, but still significantly lower than that of ^68^Ga-PSMA PET/CT. Our result also contradicted the findings reported by Zhang et al.^30^ and Zang et al.,^31^ who showed that the detection rate of ^68^Ga-PSMA PET/CT in PCa was similar to that of mpMRI in Chinese patients. This was mainly because our study assessed PCa patients with PSA levels of 4–20 ng/ml and mainly focused on intra-prostatic tumours rather than metastatic PCa lesions or patients with PSA levels ≥ 20 ng/ml. Therefore, our study is different from previous studies in terms of the methods employed and/or the patient range, and the diagnostic accuracy of ^68^Ga-PSMA PET/CT and mpMRI observed in the present study are more applicable to patient groups with low-intermediate risk.

In contrast to the organ specificity of PSA, PSMA is a transmembrane glycoprotein expressed on the cell surface of PCa cells at a much higher concentration than in normal prostate cells^11,32^, and though there were a few benign lesions of hyperplasias or inflammations, with a small number of false-positive cases, the overall image results were more theoretically reasonable than those obtained with mpMRI for detection of PCa with PSA levels of 4–20 ng/ml. Without consideration of the limiting PSA cut-off (20 ng/ml), a previous systematic review and meta-analysis by Perera et al.^14^ reported that the summary sensitivity and specificity of ^68^Ga-PSMA-11 PET/CT were both 86% in the patient-basis analysis of PCa, and the present study showed slightly higher results for sensitivity and specificity (87.88% and 88.24%). Thompson et al.^9^ and Kim et al.^10^ showed that the specificity of mpMRI was only 36–58% in PCa diagnosis, indicating a high false-positive rate, which was similar to the findings of the present study (52.94%). Although another systematic review and meta-analysis showed that the pooled average sensitivity and specificity of mpMRI for PCa detection were 89% and 73%^8^, it precisely confirmed the controversial diagnostic performance of mpMRI. These results indicated that for patients with PSA levels of 4–20 ng/ml, ^68^Ga-PSMA-617 PET/CT might possess greater ability and stability to identify benign prostatic tissue than mpMRI to avoid more false-positive cases, which might reduce overdiagnosis.

The density of PSMA receptors was reported to increase with more aggressive PCa cells^33,34^; therefore, ^68^Ga-PSMA-617 PET/CT could be used to identify higher-grade malignancies of PCa in a cohort with GS. The diagnostic accuracy of PET is known to be dependent on the harmonization of ^68^Ga-PSMA-617 PET/CT scanning and the expertise of the reporter, and on developing quantitative aids that allow the reader to determine likely clinical significance^28^. We found that the semi-quantitative SUVmax and SUVratio of lesions would be appropriate parameters to detect PCa lesions in ^68^Ga-PSMA-617 PET/CT, and were superior to the PI-RADS score in mpMRI. In our study, we found a significant correlation between higher GS of PCa lesions and higher SUVmax and SUVratio values, which is consistent with the findings of the previous study^35^. And we defined 2.040 and 4.795 as the best cut-off values for SUVmax (sensitivity 81.82%, specificity 91.18%, AUC = 0.9180) and SUVratio (sensitivity 90.01%, specificity 94.12%, AUC = 0.9608), respectively, for PCa detection. Lopci et al.^25^ reported that, for patients with overall PSA level, the ROC analysis demonstrated that a SUVmax > 4.8 could differentiate tumour lesions from benign conditions with a sensitivity of 82.4% and specificity of 72.2% (AUC = 0.843). The corresponding cut-off SUVratio was > 1.8, for which sensitivity was 94.1% and specificity was 88.9% (AUC = 0.949). The discrepancy between our own cut-off values and the values reported previously may be primarily attributable to the small sample size of the studies and to technical differences in scanners and reconstruction modalities. In comparison with a previous study, our results showed a slightly higher specificity. The reason was that the calculation in the previous study was performed in benign prostatic hyperplasia (BPH)^25^, which was characterized by slightly higher uptake values than the normal prostate corresponding to our study. Like the previous study reported^15^, our results showed that the clinical utility and benefit of ^68^Ga-PSMA-617 PET/CT was superior to that of mpMRI by decision-curve analysis, and SUVratio might be the preferred diagnostic parameter in the detection of PCa with PSA levels of 4–20 ng/ml.

In our study, the sensitivity of ^68^Ga-PSMA-617 PET/CT was relatively higher than that of ^68^Ga-PSMA-11, in comparison with previous studies^14,35,36^. Using a different radio-tracer, different patient selection criteria or randomly higher PSMA expression of the enrolled PCa patients are possible reasons. Meanwhile, the high PSMA expression of the enrolled PCa patients might be another possible reason. Immunohistochemical analysis was performed on 25 PCa tissue samples, and the PSMA-positive rate was up to 96%. Furthermore, the pathological findings of the present study indicated that all tumors were possible prostate adenocarcinomas and did not involve specific types such as neuroendocrine (< 2%) ^37^ or intraductal carcinoma (< 1%)^38^, and these special types of tumors often showed lower expression of PSMA, so ^68^Ga-PSMA-617 PET/CT examinations might be prone to false-negative results^39,40^ that would interfere with their specificity. We acknowledge that further research and analysis are required to address these issues.

There are limitations associated with this study. First, the sample size was low; although participants were selected from among 115 men, 67 patients were included in the final analysis and the number of PCa cases was 33. A larger trial is warranted. Second, the diagnosis of PCa was based on biopsy, and a benign prostate was defined on the basis of follow-up findings. Although we conducted these clinical practices according to NCCN guidelines to obtain pathological diagnosis of PCa and benign diagnosis, there were still limitations. For one, the possibilities of missed diagnoses by biopsy persisted, and considering the slow progression of PCa, the follow-up duration may not be enough, leading to the possibility of recurrence or tumorigenesis. Finally, not all the tissue samples underwent immunohistochemical analysis, which might interfere with the accuracy of differentiating histopathological subtypes and the analysis of PSMA expression.

### Conclusion

^68^Ga-PSMA-617 PET/CT imaging has better diagnostic performance, especially in terms of specificity, than mpMRI in suspected PCa patients with PSA levels of 4–20 ng/ml. Given the positive correlation between the uptake values of ^68^Ga-PSMA-617 PET/CT (SUVmax or SUVratio) and GS, ^68^Ga-PSMA-617 PET/CT could be used as an objective non-invasive imaging tool to predict PCa risk and determine the degree of malignancy. SUVratio seemed to be a more reliable diagnostic parameter of ^68^Ga-PSMA-617 PET/CT for detecting PCa with PSA levels of 4–20 ng/ml.

### Ethical approval and consent to participate

All procedures performed in studies involving human participants were in accordance with the ethical standards of the institutional and/or national research committee and with the 1964 Helsinki declaration and its later amendments or comparable ethical standards. The study was approved by the Ethics Committee of the Xijing Hospital, Fourth Military Medical University (permit: KY20162088-1). Informed consent was obtained from all participants included in the study.

## Supplementary information

Supplementary information

## Data Availability

We declare that materials described in the manuscript, including all relevant raw data, will be freely available to any scientist wishing to use them for non-commercial purposes, without breaching participant confidentiality.

## Abbreviations

^68^Ga: Gallium-68
AUC: Area under the curve
CIs: Confidence intervals
DCA: Decision curve analysis
DRE: Digital rectal examination
DWI: Diffusion-weighted imaging
GS: Gleason score
mpMRI: Multiparametric magnetic resonance imaging
NCCN: National Comprehensive Cancer Network
PCa: Prostate cancer
PET/CT: Positron emission tomography/computerized tomography
PI-RADS: Prostate imaging reporting and data system
PSA: Prostate-specific antigen
PSMA: Prostate-specific membrane antigen
RALRP: Robot-assisted laparoscopic radical prostatectomy
ROC: Operating characteristic curve
ROIs: Circular regions of interest
SUVmax: Maximal standardized uptake value
TRUSB: Transrectal ultrasound biopsy
