# Supplementary material for: Comparison of 68Ga-PSMA-617 PET/CT with mpMRI for the detection of PCa in patients with a PSA level of 4–20 ng/ml before the initial biopsy
Source: Sci Rep. 2020 Jul 3;10:10963. doi: 10.1038/s41598-020-67385-9 (PMC7334214; doi:10.1038/s41598-020-67385-9)
Supplement: Supplementary file 1 — Supplementary information [file 41598_2020_67385_MOESM1_ESM.docx]

**supplementary information**


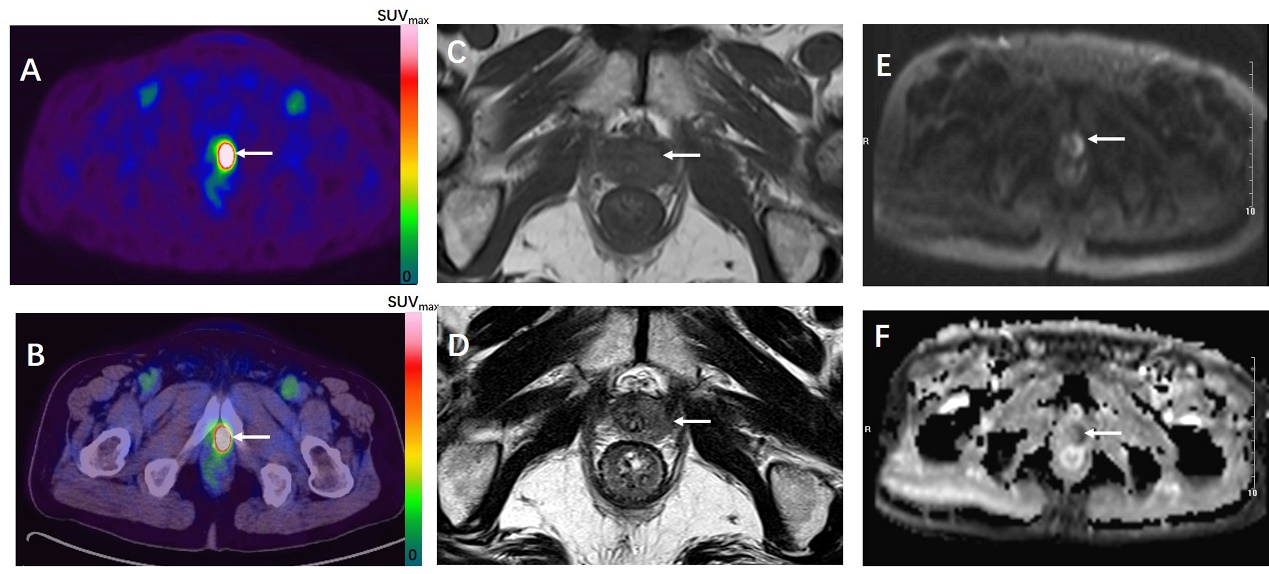
**Supplementary Figure S1.** A 70-year-old patient with PSA level of 11.52 ng/mL. ^68^Ga-PSMA-617 PET/CT imaging (A, B) showed abnormal uptake of ^68^Ga-PSMA in the left peripheral prostate zone, with an SUVmax of 17.9, suggesting PCa. In mpMRI, a signal drop in T2w (D), fast wash-out in the T1w sequence (C), focal diffusion restrictions in DWI (E), and the corresponding ADC-map (F) were interpreted as showing tumor infiltration of the left peripheral zone of the prostate. The PI-RADS score was 5. All the lesions are indicated by arrows.


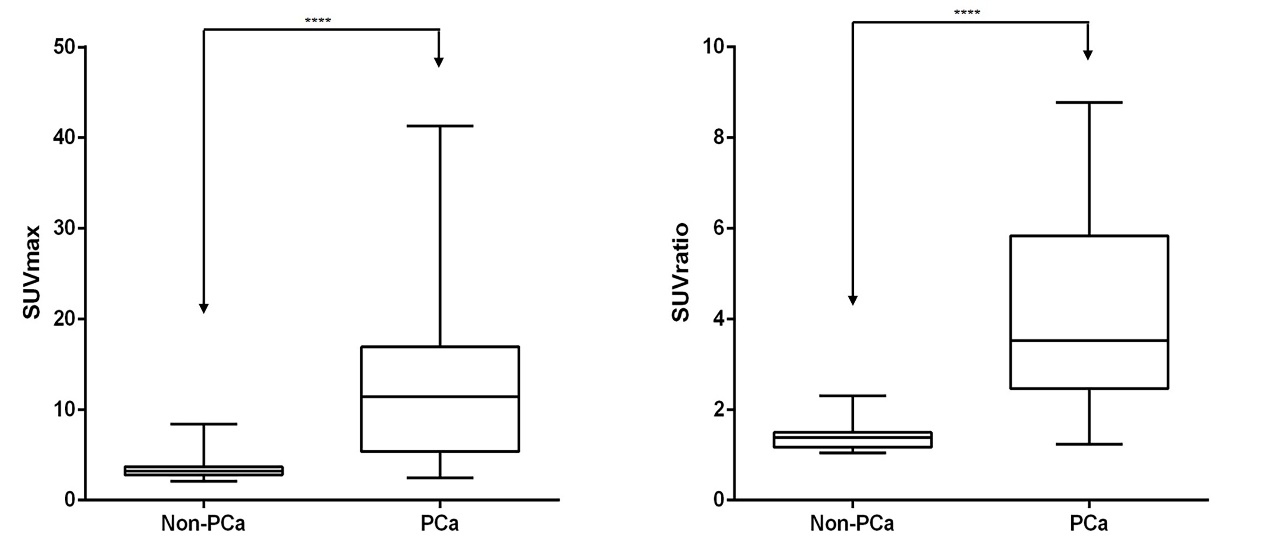
**Supplementary Figure S2.** Box-and-whisker plots for SUVmax and SUVratio based on the pathological data for PCa


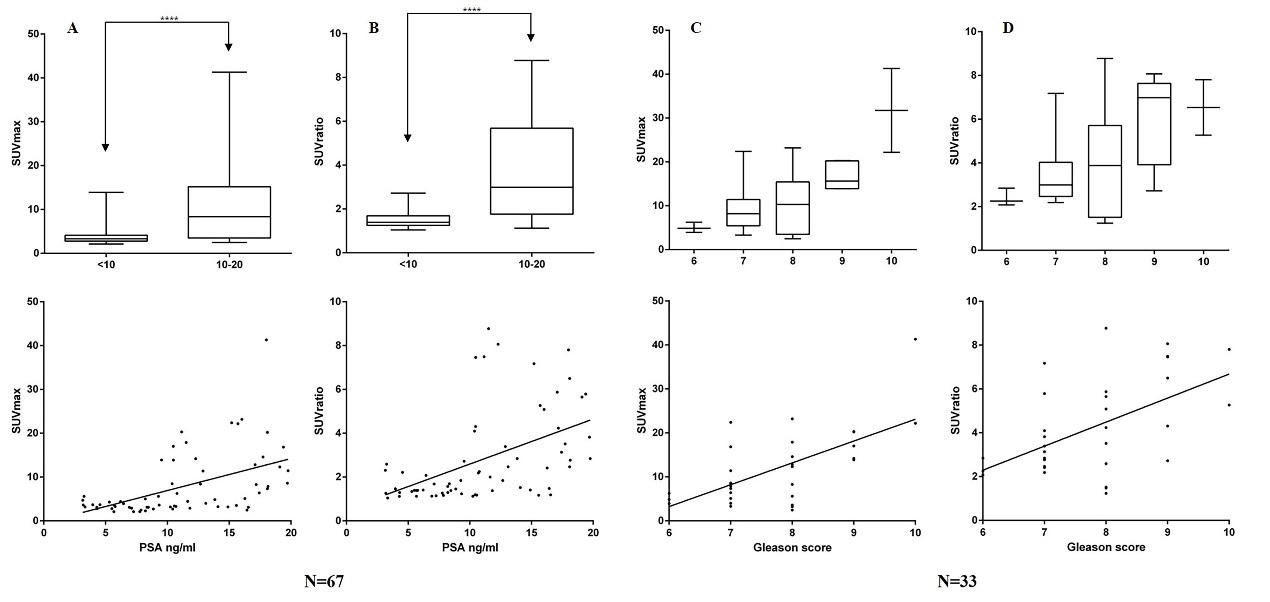
**Supplementary Figure S3.** Boxplot and linear regression of SUVmax and SUVratio according to the PSA value (A and B) and Gleason score (C and D)


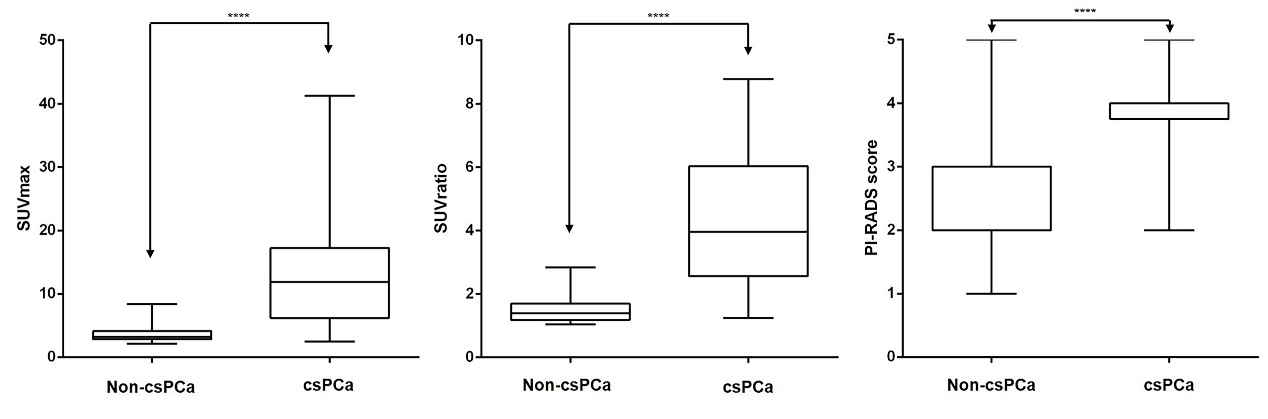
**Supplementary Figure S4.** Box-and-whisker plots for SUVmax, SUVratio, and PI-RADS score with reference to the pathology data indicating the presence of clinically significant(cs) PCa.


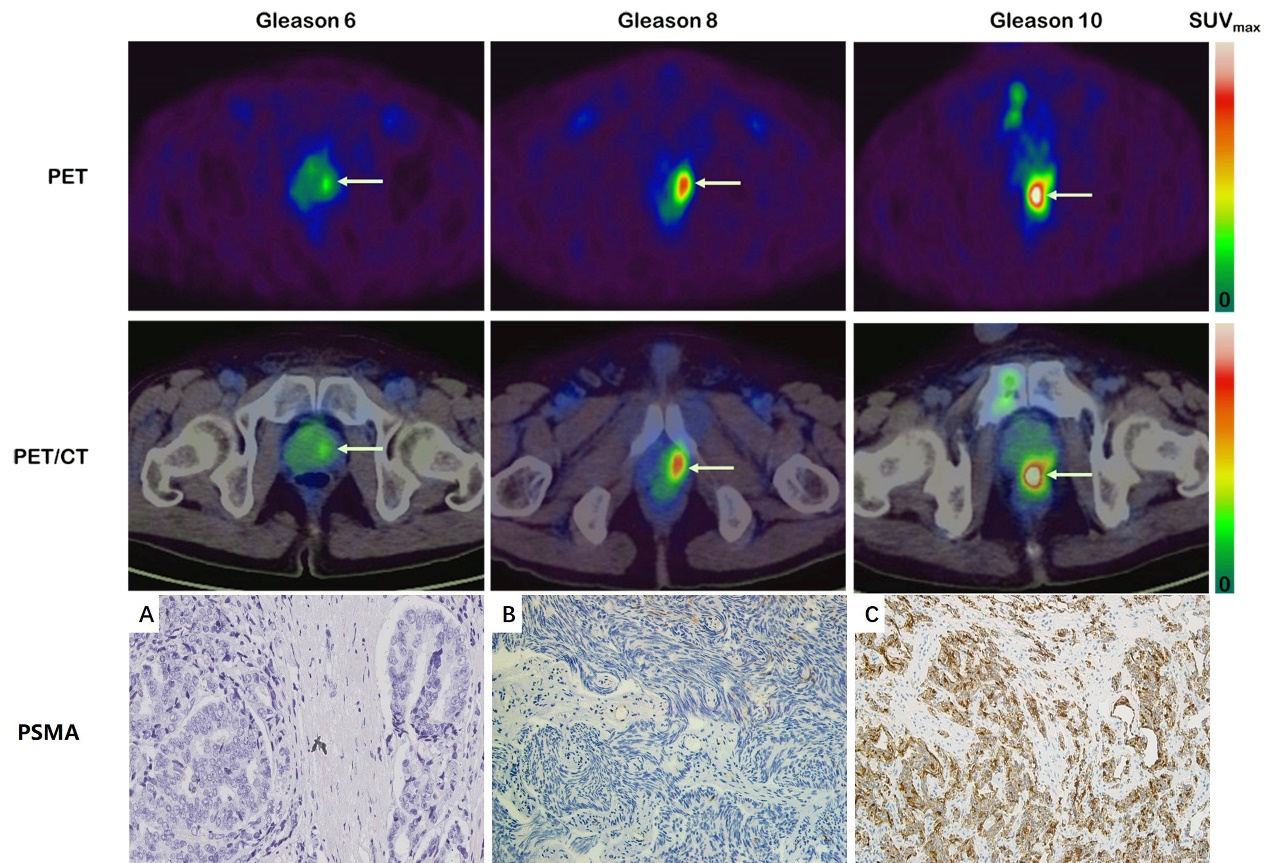
**Supplementary Figure S5.** ^68^Ga-PSMA-617 PET/CT imaging of different PCa tissues (primary lesions as indicated by arrows) and comparison of SUVmax values and Gleason scores. Immunohistochemical analysis of PSMA expression in prostate adenocarcinoma. In prostate adenocarcinoma samples, PSMA is expressed as negative (a), weakly positive (b), and strongly positive (c), corresponding to the different Gleason scores above.
